# Interventions and policy approaches to promote equity in breastfeeding

**DOI:** 10.1186/s12939-022-01670-z

**Published:** 2022-05-10

**Authors:** M. Vilar-Compte, R. Pérez-Escamilla, A. L. Ruano

**Affiliations:** 1 Department of Health, Montclair State University, University Hall 4157, 1 Normal Ave, Montclair, NJ 07043 USA; 2 Yale School of Public Health, New Haven, CT USA; 3 University of Bergen, Bergen, Norway

**Keywords:** Breastfeeding, Infant feeding, Maternal and child health, Health justice, Health inequities

Providing accessible, high-quality and equitable support for all women who want to breastfeed is a sound social investment [1]. Breastfeeding inequities are a major concern because it implies that socio-economically vulnerable women, such as women belonging to ethnic or racial groups experiencing discrimination and employed women, face major social structural barriers that prevent them from enforcing their right to breastfeed for as long as is recommended or desired [2, 3]. Given the well documented public health benefits of breastfeeding [4], this inequity can lead to increased healthcare expenditures, loses in work productivity, and poor national development since the health of tens of millions of women and their children is put at risk as a result of not being able to breastfeed as recommended [1]. Hence, fair access to breastfeeding programs guided by evidence-based policies and delivered through infrastructure that promotes, protects and support breastfeeding should be seen through a social justice lens. Any social, economic, legal, political or biomedical factor preventing women to implement their right to breastfed should be framed as a health inequity, a social injustice, and ultimately a human rights violation.

There is extensive knowledge on key obstacles preventing breastfeeding [5, 6], there is also literature suggesting that such obstacles are more salient among women with lower income levels, social positions, young age, and exposed to discrimination as a result of their race or ethnicity, country of origin, and sexual identity. Given this knowledge, this special collection brings together innovative mixed-methods research addressing the design, implementation and evaluation of programs and interventions aimed at reducing breastfeeding inequities. The aim of this special collection is to inform governments, international agencies, non-for-profit organizations and local stakeholders, and academicians about areas of action, successful interventions, and evidence feasible and sustainable designs to promote, protect and support breastfeeding through a social justice lens.

The collection brings together evidence from 17 countries from diverse regions of the world (see Fig. 1), as well as, from three global studies [7–9]. The studies represent countries with different income levels and address issues across all the layers of the socioecological model (see Fig. 2). The studies also enrich the field through innovative methodological approaches exemplified by the use of diverse qualitative and quantitative designs, mixed-methods, economic analyses, and implementation science perspectives. In this introduction to the special collection, we map out and connect the studies based on the socioecological model, which is fundamental for understanding and addressing breastfeeding inequities.

**Fig. 1:**
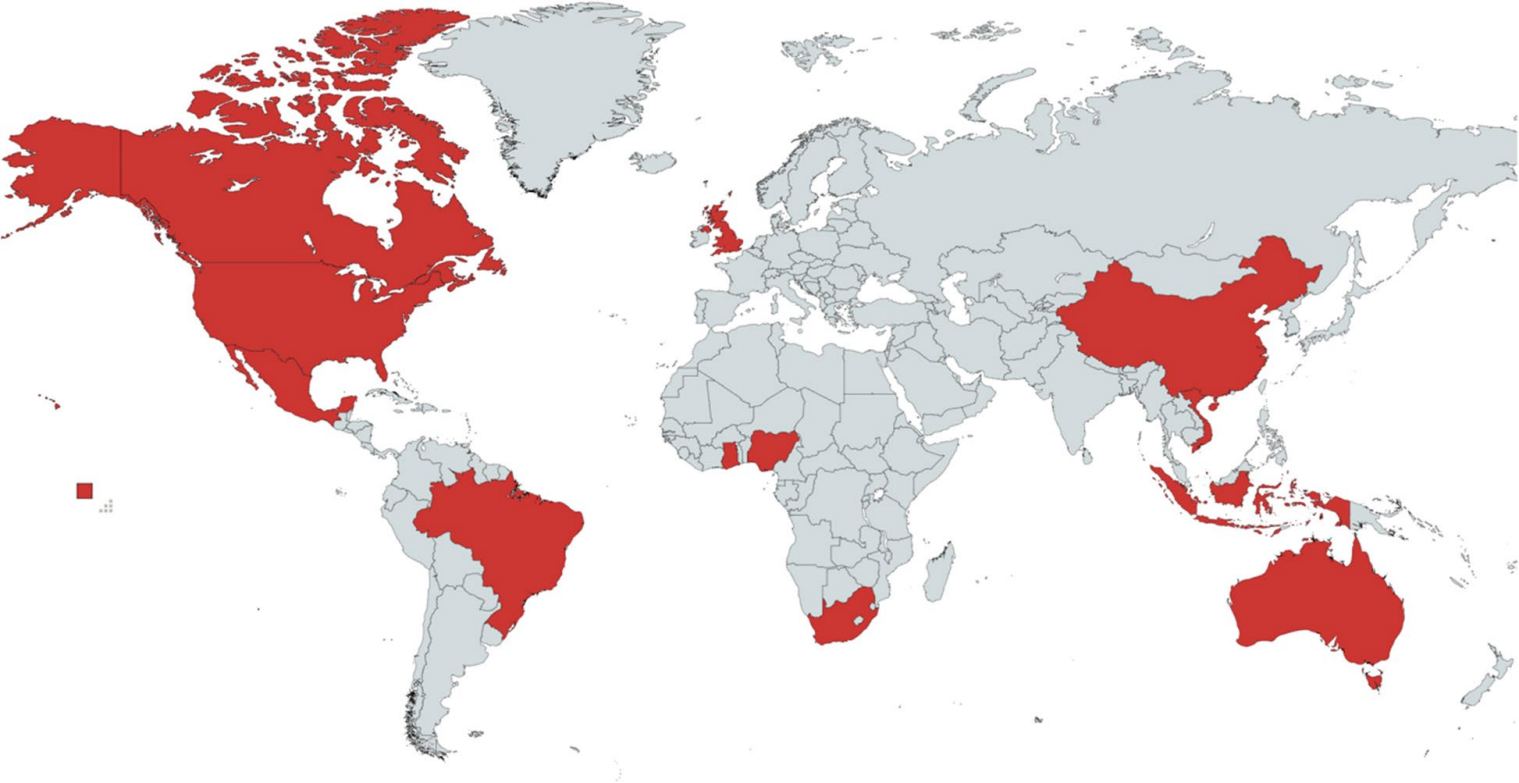
Geographic distribution of the studies included in the Special Collection on breastfeeding inequities

**Fig. 2:**
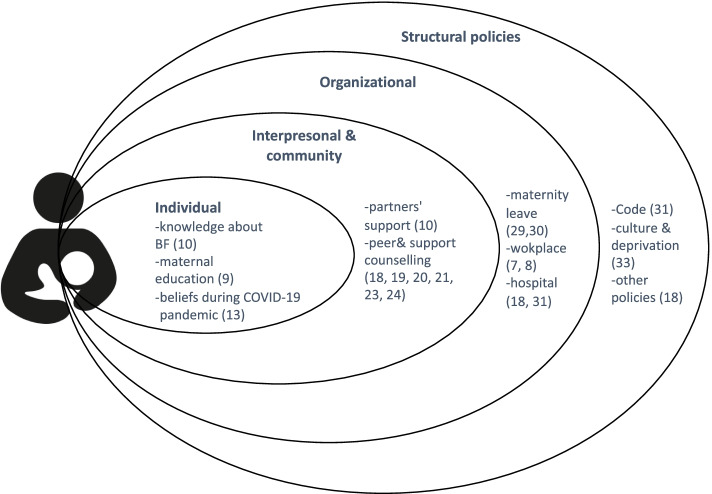
Interventions and evidence addressed in the special collection by socioecological layer

## Evidence and interventions at the individual level

Socioeconomic inequities are manifested in differential exclusive breastfeeding (EBF) behaviors and corresponding ideation factors. Abegunde et al. [10] explore inequities in the practice of EBF in Nigeria and its association with psychosocial factors through a ‘concentration index method’, which allowed decomposing the contributions from multiple determinants on EBF behaviors. This novel approach in breastfeeding analysis estimated avoidable inequities in EBF and redistribution that could mitigate them. Findings suggest that the inequality in the distribution of EBF behavior is avoidable. Specifically, receiving at least four antenatal visits during pregnancy, and having partners be supportive of women’s decision to breastfeed exclusively and that knew the benefits from EBF, were protective factors for EBF. Hence, the findings suggest that improving and expanding antenatal care coverage through pro-poor intervention could improve breastfeeding practices among the socioeconomically disadvantaged groups. From a socioecological approach, this study addressed individual level and interpersonal relationships and how these can be targeted through interventions at the institutional level (i.e. antenatal care).

Education level is a known factor affecting breastfeeding practices. In low- and middle- income countries (LMICs) breastfeeding is more prevalent among women with lower levels compared to women with higher levels of formal education [11]. Despite the undisputable relevance of increasing the levels of formal education to empower girls and young women in LMICs, this might negatively impact breastfeeding rates, hence, policies and programs should consider how to protect breastfeeding while promoting gender equity through education. It is important to understand rates of change in breastfeeding outcomes across time and population subgroups. Neves et al. [9] address this issue by analyzing breastfeeding indicators and use of breast milk substitutes for infants and young children by level of maternal education in 81 LMICs. Their study suggest that the rate of decline is faster in several optimal breastfeeding outcomes among women with no formal education compared to women with primary and secondary or higher education. This is a concerning breastfeeding inequity trend as children of women with no formal education living in LMICs are generally at higher risk of morbidity and mortality of infectious diseases that breastfeeding could prevent.

The collection clearly illustrates how socioeconomic disadvantages can magnify breastfeeding inequities during crises including public health emergencies. As the evidence became available, clear World Health Organization (WHO) guidelines recommended that women with COVID-19 should breastfeed their babies [12] and that feeding directly from the breast should be supported as the preferred recommended infant feeding option during the pandemic. In spite of this, the study by Vilar-Compte et al. [13] highlights that adults in Mexico living in households with children under 3 years of age faced harmful disinformation advising mothers to not breastfeed if they were infected with COVID-19. The study documented that the belief about the need for separating the dyad due to COVID-19 was more prevalent among respondents with a lower socioeconomic status. It is likely that the evidence-based recommendations with the correct information were disseminated through media platforms that are less commonly accessed by the lower socioeconomic levels. Hence this study provides valuable evidence on the need to ensure that lower socio-economic groups are effectively reached with sound breastfeeding recommendations during public health emergencies.

## Interpersonal and community level interventions

The 2016 Lancet Early Childhood Development Series highlighted the need for nurturing care in early childhood, including responsive parenting and feeding [14]. Breastfeeding in the context of responsive feeding is fundamental for proper lifetime health and development and can therefore be a key piece in addressing equity and social justice [15]. Breastfeeding interventions in the context of responsive feeding require considering the mother-child dyad and how it is nested in the family context. Shu et al. [16] present a research protocol of a multi-center longitudinal study in China that will address how suboptimal breastfeeding can shorten the duration of the breastfeeding itself and affect the infant’s growth and development indicators. While accounting for mothers’ responsive feeding behaviors and feeding beliefs, as well as parental factors such as stress and household characteristics, this study will generate much needed knowledge to design equity enhancing maternal-child interventions based on the nurturing care framework.

Breastfeeding peer counseling involves the provision of breastfeeding education and support by community health workers known as peer counselors. It is embedded in a nurturing care perspective, and is a proven way to promote and support breastfeeding among low-income minority women [17]. The systematic review by Segura-Pérez et al. [18] in this collection, provides strong evidence about the central role that peer counseling programs must play to advance breastfeeding equity among women belonging to ethnic or racial groups that have been historically discriminated. Rhodes et al. [19] describe the process of designing, and the impact on breastfeeding outcomes of the Breastfeeding Heritage and Pride program, an innovative evidence-based breastfeeding peer counseling program available for low-income minority women in the United States. The experience of this program offers key lessons about how to co-design and implementation effective breastfeeding peer counselling programs. Effective approaches should be based on community-engaged formative research, as well as proper training and retention of peer counselors who are empowered through supportive supervision from certified breastfeeding consultants or specialists. As shown in this study, peer counselors should have the capacity to follow and support women through pregnancy and postpartum both through community and clinical settings. In addition, the paper emphasizes the central relevance of monitoring and evaluation systems to support continuous quality improvement in counselling interventions. As highlighted by Rhodes et al. [19], postnatal lactation support services, are easier to support and sustain if they are embedded or linked to already existing maternal-child nutrition programs or healthcare services. Francis et al. [20] provide further evidence in this respect by describing infant feeding practices and service use of a postnatal lactation support initiative for vulnerable mothers enrolled in the Canada Prenatal Nutrition Program. Findings from one site in Toronto indicated a high uptake of the lactation support program by the target women. While women in the program experienced multiple vulnerabilities known to be associated with breastfeeding, the authors found a strong adherence to many recommended infants feeding practices, including breastfeeding initiation, and continued breastfeeding at 6 months. While these findings are promising, it is always relevant to consider the feasibility and adaption factors for further scale up, in this sense, implementation science will be an important tool to use. The study by Hunt et al. [21] contributes to this knowledge by exploring through a case study qualitative approach how United Kingdom non-for-profit breastfeeding organizations have developed breastfeeding peer support services in areas of deprivation. While women appreciated the practical, emotional and informational support of peer support interventions, one of the key findings of the study was that while non-for-profit organizations seek to enable women’s access to individual, social and community supportive breastfeeding environments, their needs are not always considered in the service development. Therefore there is a need to take into account contextual issues when conducting community engaged program co-design that effectively meets the community needs and wants [22].

This collection clearly highlights that interpersonal counseling has mainly been proven to be an efficient breastfeeding intervention among vulnerable women living in high income settings, but evidence from LMIC is still scarce. Bueno-Gutiérrez et al. [23] fill this gap by evaluating the effects of interpersonal counseling on EBF based on a prior formative research to co-design and implement a breastfeeding support program delivered by trained nurses thorough a primary health center serving a low-income uninsured population in Mexico, a middle-high income country. Findings suggest that counselling vulnerable women had a positive effect in decreasing negative attitudes towards breastfeeding and on EBF; hence, these encouraging outcomes were similar to those reported in high-income countries. In addition, the study identified multiple obstacles that women faced in achieving their breastfeeding goals or intentions including unsupportive environments for breastfeeding in public, breast pain, self-reported milk insufficiency, and return to the workplace, which suggest that counseling interventions should target challenges that inform different layers of the socioecological model. Further evidence about the application of peer counseling in LMICs is provided by Nguyen et al. [24] who examined a community peer counseling mother support model implemented in remote villages of Vietnam. They found that in rural areas of this low-middle income country, community peer counseling plausibly contributed to improve early breastfeeding and EBF practices. The intervention was integrated into the existing health care system, which is fundamental for program scale up and sustainability. It is noteworthy that this model was found to be financially feasible, as reaching women an average of 5 times during the first 1000 days of life was associated with an average cost of US$15 per client.

## Organizational level interventions: hospital and workplace

In the last decades, the number of women entering the labor force has steadily increased and women are representing a larger share of the labor market than ever before [25]. Unfortunately, return to work continues to be a strong barrier to breastfeeding, which makes maternity protection policies and programs fundamental to advance breastfeeding globally. Maternity mandates are key community and organizational level policies designed to promote optimal maternal-infant health and nutrition, and gender equity [26]. However, such mandates focus only on women employed in the formal economic sector [27]. This is worrisome as women make up a disproportionate percentage of employees in the informal sector, especially in LMICs [28]. Therefore, there is strong justification to mandate maternity benefits for women employed in the informal sector but how to do so remains a complex challenge that requires shorter-term innovative and pragmatic approaches. In this collection, two papers explore the cost of implementing a maternity cash transfer [29, 30] in three different countries (Brazil, Ghana, and Indonesia) among women employed in the informal economy. Findings suggest that implementing such intervention would take a negligible percentage of the gross domestic product (GDP) - below 0.8% in all cases -, and such cash transfer could increase the chances that a working woman employed in the informal sector could ensure a basic level of income for her and her family empowering women to stay home with their babies without facing a loss of income. This could have further long-term benefits including improved health and nutrition for the infant, human capital development, gender equity, and national development. Keeping in line with the inclusion of innovative methodological approaches in this collection, Siregar et al. [30] performed and compared the financial estimations for maternity leave benefits for women working in the informal sector in Indonesia using two different costing approaches, and demonstrating that similar findings were obtained regardless of methodology used. It is reassuring that reliable methods are now available to cost out maternity benefits for highly vulnerable women. However, at the end of the day implementing a maternity cash transfer will require strong political will and the engagement of multiple decision makers and institutions taking contextual factors into account. Cost analyses, such as the ones included in this collection, are needed to advocate for the necessary budgetary resources to implement and sustain equitable breastfeeding protection, promoting, and support policies and interventions [18].

In addition to maternity mandates, effective workplace breastfeeding interventions and programs are needed. Policies at the workplace level are fundamental for gender equity, equitable work environments and fair start in life for infants and young children of working mothers [8]. This collection includes two papers addressing workplace interventions. The first, is a global systematic review [8], which concludes that workplace interventions are likely to help increase the duration of breastfeeding and to prevent the early introduction of breast milk substitutes, a practice that strongly undermines breastfeeding. Interventions such as worksite lactation spaces, breastmilk extraction breaks, and supportive organizational polices were identified as key strategies to achieve more equitable conditions for breastfeeding among employed mothers. However, the review also suggested that for effective and sustainable interventions, multilevel strategies are needed considering individual, interpersonal and organizational factors, as posited by the social-ecological model. The second study [7] is a realistic review that included evidence from 11 countries. It seeks to parse out how workplace interventions work, and how their influence on breastfeeding may differ across contexts. This review provides crucial evidence to operationalize, implement, and disseminate robust and effective worksite lactation programs. Findings from this review were integrated into a context-mechanisms-outcome framework that allowed for identifying potential mechanisms that might help understanding how workplace interventions influence breastfeeding outcomes. The three mechanisms highlighted by the review were time to breastfeed during work; awareness of pro-breastfeeding interventions in the workplace; and a change in culture and physical environment in the workplace. This framing is highly consistent with the systematic review by Vilar-Compte et al. [8], indicating that a socioecological approach is needed to promote a change in breastfeeding habits at the workplace. In addition, and in full consistency with the maternity benefits literature, almost none of the studies included on the realist review focused on women employed in the informal sector. This is an indication of a profound inequity in the focus for researchers leaving out an area that requires an urgent call for action.

Maternity services embedded in complex health care systems are fundamental for advancing breastfeeding on large scale. The WHO and UNICEF launched the Baby Friendly Hospital Initiative (BFHI) in 1991 to incentivize maternity services to implement the Ten Steps to Successful Breastfeeding (Ten Steps). Segura-Pérez et al. [18] review documents in their systematic review reporting that maternity care practices consistent with the Ten Steps improve breastfeeding outcomes among women in the United States belonging to ethnic and racial groups that have been historically discriminated. The findings indicate that BFHI can contribute to decreasing breastfeeding inequities, keeping in mind cross-cultural training for maternity care staff is needed to effectively support socio-economically vulnerable women. Despite these potential benefits, the uptake of BFHI globally has been slow. In order to assess incentives for BFHI accreditation, Pramono et al. [31] assess the social return of investment of maintaining the BFHI accreditation in a public maternity unit in Australia. Social return of investment is another novel methodology included in this collection, based on monetizing social impacts with inputs from diverse stakeholders. Pramono et al. [31] estimate that the social return of investment ratio in the analyzed unit was approximately AU$ 55:1, which means that for every AU$1 invested in maintaining BFHI accreditation the facility generated approximately AU$55 of benefit. This suggest that scaling up BFHI accreditation can be an effective public health intervention and a sound investment from a societal perspective. The perceived lack of policy commitment to BFHI globally might be due to low valuation of breastfeeding due to its intangible contributions from a main economic perspective, combined with aforementioned lack of equitable community engaged co-design and co-implementation practices. Social return of investment estimations are needed to help inform policymakers about the need to refocus attention in implementing the Ten Steps to Successful Breastfeeding through an equity lens.

## Structural policies

This collection highlights the relevance and existing challenges in implementing structural policies needed to reduce breastfeeding inequities. Vitalis et al. [32] document the experience of South Africa on how the initial WHO infant feeding recommendations for HIV positive women ended up negatively affecting breastfeeding practices also in HIV negative women and introduced major challenges to the enforcement of the International Code of Marketing of Breast-milk Substitutes (The Code). Through a scoping review Vitalis et al. [32] highlighted that maternal HIV, which usually coexists with poverty became a powerful syndemic, as breastfeeding was disincentivized in the early WHO guidelines issued in the context of the then lack of access to effective antiretroviral treatments. Despite the enormous improvements in access to antiretrovirals as time went by in South Africa, and the issuing of new guidelines by WHO supporting breastfeeding in this new context, the legacy of the initial recommendations continues to be a formidable challenge for South African efforts at enforcing the Code. Key challenges that are still leading to major breastfeeding inequities include social norms and medical practices endorsing breast milk substitutes over breastfeeding, and widespread marketing of breast milk substitutes that violate the Code. This study highlights why exploring mothers’ experiences of infant feeding decisions and the barriers toward accessing infant feeding services often implies looking at how historical trauma, cultural norms, deprivation and marginalization, and inequitable access to services work together in synergy towards adding barriers at different levels of the socioecological framework that lead to breastfeeding inequities. In this vein, Cook et al. [33], conducted a qualitative study, to assess what influences feeding methods decisions, including services accessed by women of a deprived and culturally diverse community in the United Kingdom. Self-report of milk insufficiency was a major factor to introduce breastmilk substitutes, which might result from a lack of adequate strategies and services to reassure and support women since the beginning and throughout their breastfeeding journey. When available, breastfeeding support services were linked to increased confidence and motivation of breastfeeding, and mothers valued the practical support around factors such as positioning and attachment techniques. However, such services need to be culturally sound, address different acculturation processes among immigrants and cultural conflicts among vulnerable populations of diverse social and national origins.

Lastly and fully consistent with the social-ecological model, it is also fundamental to recognize the role that broader polices and laws can have in protecting, promoting and supporting breastfeeding. In the systematic review of breastfeeding interventions among women belonging to ethnic and racial groups historically discriminated in the United States, Segura-Pérez et al. [18] document the effectiveness of some structural policies, including mandated coverage of support services under the Affordable Care Act, and state-level maternity protection laws such as those promoting breastfeeding at the workplace, permitting breastfeeding in any public or private area, exempting breastfeeding women from jury duty, and enabling breastfeeding awareness campaigns. Favorable breastfeeding outcomes were also associated with federally funded programs, particularly the Supplemental Nutrition Program for Women, Infants and Children’s (WIC’s). This key review highlights the role that policymakers can have in enabling and promoting breastfeeding friendly environments, a crucial step needed to benefit the most vulnerable mothers and their infants in highly inequitable societies across the world.

## Conclusions

As highlighted by several authors in this special collection, combining interventions at different levels of the socioecological model is strongly recommended as it would address the different health and social environments necessary for equitable and successful breastfeeding. Future studies and interventions would also benefit from using implementation science frameworks and theories to address structural inequities such as class oppression, racism, and discrimination [22]. In designing, implementing, and sustaining interventions to reduce breastfeeding inequities, behavioral science research and the co-design of programs based on community needs- and wants will allow to more successfully protect, promote, and support, the right that women have to breastfeed for as long as is recommended or desired.

## Data Availability

Not applicable.

## Abbreviations

EBF: Exclusive Breastfeeding
LMICS: Low- And Middle-Income Countries
WHO: World Health Organization
UNICEF: United Nations International Children’s Emergency Fund
BFHI: Baby Friendly Hospital Initiative
GDP: Gross Domestic Product
WIC: Supplemental Nutrition Program for Women, Infant and Children
